# DNA Methylation: A Key Regulator in Male and Female Reproductive Outcomes

**DOI:** 10.3390/life15071109

**Published:** 2025-07-16

**Authors:** Adedeji O. Adetunji, Henrietta Owusu, Esiosa F. Adewale, Precious Adedayo Adesina, Christian Xedzro, Tolulope Peter Saliu, Shahidul Islam, Zhendong Zhu, Olanrewaju B. Morenikeji

**Affiliations:** 1 Department of Agriculture, University of Arkansas at Pine Bluff, Pine Bluff, AR 71601, USA; owusuh8210@uapb.edu (H.O.); islams@uapb.edu (S.I.); 2 Department of Biology, University of Louisville, Louisville, KY 40292, USA; esiosa.adewale@louisville.edu; 3 National Center for Advancing Translational Sciences, Division for Pre-Clinical Innovation, National Institutes of Health, Bethesda, MD 20850, USA; precious.adesina@nih.gov; 4 Laboratory of Food Microbiology and Hygiene, Hiroshima University, Higashihiroshima 739-8528, Japan; 5 Department of Physiology, College of Medicine, University of Kentucky, Lexington, KY 40536, USA; tpsa222@uky.edu; 6 College of Animal Science and Technology, Qingdao Agricultural University, Qingdao 266109, China; zzd2020@qau.edu.cn; 7 Department of Biological and Health Sciences, University of Pittsburgh, Bradford, PA 16701, USA; obm3@pitt.edu

**Keywords:** DNA methylation, epigenetic modification, age, infection, steroids, lifestyle, reproductive health, obesity, stress

## Abstract

DNA methylation is a well-studied epigenetic modification that regulates gene expression, maintains genome integrity, and influences cell fate. It is strictly regulated by a group of enzymes known as DNA methyltransferases (DNMTs). Most DNA methylation occurs at cytosines within symmetrical CpG dinucleotide base pairs, often located at gene promoters or other regulatory elements. Thus, methylation of a promoter CpG island leads to stable transcriptional repression of the associated gene. Nonetheless, abnormal gene expression caused by alterations in DNA methylation has been linked to infertility in both males and females, as well as to reproductive potential and improper post-fertilization embryo development. Recent epigenetic advancements have highlighted the significant association between epigenetic modification and reproductive health outcomes, garnering considerable attention. In this review, we explore significant advancements in understanding DNA methylation, emphasizing its establishment, maintenance, and functions in male and female reproductive sex cells. We also shed light on the recent discoveries on the influence of environmental exposures, nutrition, infection, stress, and lifestyle choices on DNA methylation. Finally, we discuss the latest insights and future directions concerning the diverse functions of DNA methylation in reproductive outcomes.

## 1. Introduction

DNA methylation is a heritable epigenetic modification that plays a central role in regulating gene expression, maintaining genome stability, and guiding cell lineage commitment during development [1,2]. In animals, its importance is particularly evident in the reproductive system, where precise methylation patterns are required for germ cell specification, meiotic progression, and epigenetic reprogramming [3,4,5,6]. Although CpG methylation has long been the primary focus of epigenetic research, recent studies have revealed that non-CpG methylation (particularly at CpA, CpT, and CpC sites) and 5-hydroxymethylcytosine (5hmC) are also dynamically regulated during germline development. In male prospermatogonia, non-CpG methylation accumulates asymmetrically during mitotic arrest and is later lost upon mitotic resumption, while in female oocytes, non-CpG methylation increases progressively during growth, coinciding with imprint establishment [7]. Similarly, 5hmC exhibits developmental stage-specific enrichment in both embryonic and germline cells, suggesting potential roles in epigenetic reprogramming and sex-specific germ cell maturation [8].

Epigenetic regulation in the germline is highly dynamic, characterized by two major waves of genome-wide reprogramming: the first occurring shortly after fertilization during early embryogenesis, and the second taking place in the developing primordial germ cells (PGCs) during gametogenesis [3,4,9,10]. Each wave involves extensive DNA demethylation followed by remethylation, but with distinct biological purposes. The initial wave erases most parental methylation marks to re-establish a pluripotent state in the early embryo, preparing cells for lineage commitment. In contrast, the second wave, which unfolds as PGCs migrate and colonize the gonads, resets the epigenome again; including imprinted regions and culminates in the establishment of sex-specific methylation landscapes critical for germ cell identity and function. Disruptions in either phase can lead to impaired gametogenesis, infertility, or transgenerational epigenetic errors [9,10,11]. Central to these processes are the DNA methyltransferases (DNMTs): DNMT3A and DNMT3B function as de novo methyltransferases, responsible for establishing new methylation patterns during embryogenesis and germ cell development [12], while DNMT1 maintains these patterns across cell divisions by replicating parental DNA methylation patterns onto newly synthesized DNA [12,13]. Although catalytically inactive, DNMT3L plays a crucial supporting role by stimulating the enzymatic activity of DNMT3A and DNMT3B in the germline and is indispensable for the establishment of parental imprints [1,14].

**Figure 1:**
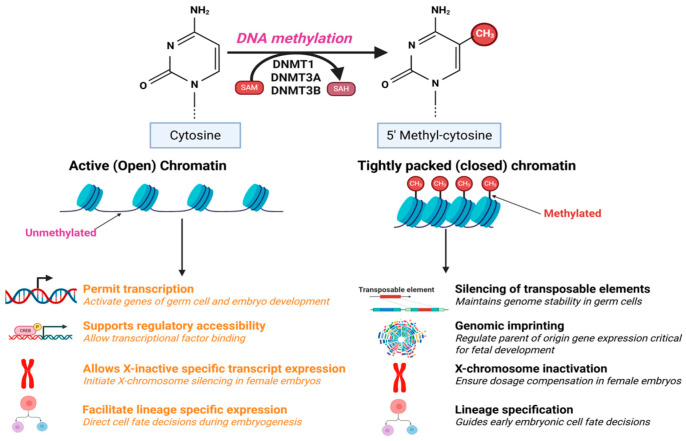
Functional roles of DNA methylation during gametogenesis and early embryonic development. DNA methylation, catalyzed DNMTs, modulates chromatin structure by promoting the transition between active (open) and repressive (closed) states. This schematic illustrates how methylation-dependent chromatin remodeling coordinates essential developmental processes, including transcriptional regulation, transposable element silencing, genomic imprinting, X-chromosome inactivation, and lineage specification. Arrows emerging from open chromatin denote processes that require transcriptional accessibility, whereas those from closed chromatin indicate pathways reliant on gene silencing. Together, these mechanisms underpin genome stability, reproductive fitness, and the faithful transmission of epigenetic information.

Importantly, CpG and non-CpG methylation are differentially regulated across germline development. CpG methylation is faithfully propagated during cell division by DNMT1, ensuring mitotic inheritance of epigenetic patterns [15]. In contrast, non-CpG methylation, particularly at CpA sites, emerges during the mitotic arrest of male germ cells and is progressively lost upon resumption of proliferation [8]. The accumulation of non-CpG methylation in male germ cells is not a passive consequence of DNMT expression but reflects a finely regulated balance between de novo methylation activity and cell cycle dynamics [8]. In female germ cells, both CpG and non-CpG methylation accumulate during oocyte growth, contributing to the establishment of genomic imprints and preparing the oocyte for fertilization and early embryogenesis [16]. The preservation of these epigenetic patterns across generations is supported by accessory factors such as UHRF1, which recognizes hemimethylated CpG sites and recruits DNMT1 through interactions with repressive histone modification [17,18]. Notably, this intricate regulatory network is shaped not only by intrinsic developmental programs but also by extrinsic cues. Environmental factors including nutrition, stress and toxicant exposure have been shown to alter DNA methylation landscapes in germ cells [19,20]. Such perturbations can impair reproductive competence and may be transmitted across generations, positioning DNA methylation as a central molecular interface between environmental signals and heritable reproductive outcomes [20] (Figure 1). Here, we review recent advances in the understanding of DNA methylation’s establishment, maintenance, and functional roles in male and female germ cells. We examine emerging insights into the dynamic patterns of DNA methylation and the molecular mechanisms governing its regulation during germline development. We further discuss the implications of altered DNA methylation in reproductive outcomes, considering influences such as aging, infection, pharmaceuticals, steroids, stress, and lifestyle factors. Finally, we highlight key areas for future investigation, aiming to clarify how DNA methylation integrates intrinsic and environmental signals to shape reproductive health across diverse contexts.

## 2. Role of DNA Methylation in Transcription

In the context of the role of DNA methylation in transcription and repression, evidence suggest a correlation between DNA methylation and gene silencing, which increases with the density of CpG dinucleotides at the promoter regions [21,22]. However, the precise mechanisms by which this process leads to transcriptional inhibition remain unclear, as the methylation mark itself does not appear to directly induce or confer silencing. Regions characterized by accessible chromatin typically exhibit low levels of methylation or are completely unmethylated, indicating a mutually exclusive relationship between the binding of transcription factors and DNA methylation [23]. A recent study conducted by Yin et al. found that specific transcription factors are sensitive to CpG methylation. Among the 519 classified human transcription factors, only 60% were capable of binding to one or more enriched sequences whose enrichment was influenced by CpG methylation. Notably, 117 (23%) of these transcriptional factors demonstrated a reduction in binding capacity to their motifs when methylated, in contrast to unmethylated counterparts [24]. This confirms the significant role of DNA methylation in regulating gene expression during mammalian development. By preventing the binding of such transcription factors, DNA methylation can impair transcriptional activation of certain promoters containing their sequence-recognition motifs. Additionally, methylated cytosines can also act as binding sites for transcription activators. Most DNA methylation occurs at cytosines within symmetrical CpG dinucleotide base pairs which are often located at gene promoters or other regulatory elements. Thus, methylation of a promoter CpG island leads to stable transcriptional repression of the associated gene [25]. In embryonic stem cells, certain promoter silencing is facilitated by the deposition of histone H3 lysine 27 tri-methylation (H3K27me3) via Polycomb repressive complex 2, a more adaptable mode of silencing compared to DNA methylation [12,26], suggesting the critical role of DNA methylation in regulating gene expression during mammalian development.

DNA methylation also plays an essential role in the formation of heterochromatin, orchestrated by the recruitment of chromatin remodelers and modifiers through the activity of DNMT proteins, de novo DNMTs function in conjunction with the chromatin remodeler lymphocyte-specific helicase, as well as H3K9 methyltransferases and histone deacetylases [14,27,28,29]. Nevertheless, there are three primary classes of genes in which stable, lifelong DNA methylation-based silencing in somatic tissues is essential: germline-specific gene, imprinted genes, and genes on the inactive X chromosome. For X-chromosome inactivation especially in female mammals, one X chromosome in each cell is randomly silenced by the activity in *cis* of the non-coding RNA X-inactive specific transcript. In this process of X-chromosome inactivation, DNA methylation of X-linked CpG islands (CGIs) appears to occur relatively late to function as a final lock added after the genes have already been silenced [21]. In the context of genomic imprinting, DNA methylation is established differentially in the two parental germlines. These imprinted patterns can endure the genomic reprogramming of DNA methylation that occurs in the early stages of embryogenesis. Approximately 20 genomic regions in humans, known as imprinting control regions (ICRs), resist this reprogramming and enforce monoallelic expression of adjacent genes. Also, gene clusters of specific chromosomal regions are coordinately silenced in imprinting through the methylation of an imprinting center—also referred to as differentially methylated regions (DMRs)—that frequently overlap with CpG islands. It is important to recognize that the expression of DMRs in the oocyte may facilitate their subsequent CpG modification by preserving an open chromatin structure that is accessible to de novo methylation [30]. As an epigenetic modification, DNA methylation is recognized for its stability and heritability. Global alterations in DNA methylation profiles typically occur during specific phases of genome-wide reprogramming, particularly in preimplantation embryos and primordial germ cells. During these critical stages, methylation marks are removed and reestablished, leading to the formation of new epigenotypes that facilitate new cellular functions. Recent advances in microarray and sequencing technology, such as high-throughput sequencing of bisulfite-treated DNA (bisulfite-seq) and the immunoprecipitation of methylcytosine-containing DNA, together with enhanced bioinformatic capabilities, have enabled comprehensive analysis of these dynamic methylation patterns [31,32].

## 3. Role of DNA Methylation in Male and Female Reproductive Gamates

DNA methylation is significantly reprogrammed in both males and females during gametogenesis to establish sex-specific epigenetic marks essential for normal reproductive function. Methylation marks regulate gene expression in gamete formation and embryogenesis with relevance to cell fate determination and developmental processes [33,34]. During gametogenesis, DNA methylation is more dynamic than in somatic cells and also plays uniquely critical regulatory roles essential for proper germ cell development. In primordial germ cells (PGCs), a comprehensive demethylation phase (Embryonic days 8.5–13.5 in mice) resets epigenetic information via both passive and active TET-mediated routes [35]. This clearing is followed by sex-specific de novo remethylation, orchestrated by DNMT3A/B and the adaptor DNMT3L, which is critical not only for re-establishing genomic imprints, but also for silencing transposable elements that threaten genome integrity [3]. In mice, Dnmt3l knockout leads to loss of methylation in both CG and CH contexts, failure to silence retrotransposons, disrupted spermatogenesis, and complete infertility [36]. Precise DNA methylation during gametogenesis safeguards totipotency after fertilization, secures imprinting patterns, and protects the genome, highlighting why it is more pivotal during gametogenesis than in any other developmental context.

Besides, recent advances have expanded this canonical framework by uncovering significant roles for non-CpG methylation (mCH, where H = A, T, or C) and 5-hydroxymethylcytosine (5hmC) in germ cell epigenetics and early embryonic reprogramming [7,8,37]. Non-CpG methylation refers to the addition of methyl groups to cytosines outside the typical CpG dinucleotide context. This modification is deposited primarily by the de novo DNMT3A and DNMT3B, enzymes responsible for establishing new methylation patterns during development [7,12]. Non-CpG methylation is particularly enriched in oocytes and pluripotent cells, where it correlates with active gene transcription and confers epigenetic plasticity, enabling cells to respond flexibly to developmental cues [38,39,40]. In male prospermatogonia, non-CpG methylation accumulates during mitotic arrest; a phase when cells temporarily stop dividing, and is progressively lost upon cell cycle re-entry, reflecting a tightly regulated interplay between methyltransferase activity and cell proliferation [8]. In female gametes, the enrichment of non-CpG methylation within gene bodies suggests a functional role in modulating gene expression programs that prepare the oocyte for fertilization and early embryonic development [41]. These mechanisms have been comprehensively reviewed, highlighting non-CpG methylation as a dynamic and cell type–specific epigenetic feature integral to mammalian development [42]. Concurrently, 5-hydroxymethylcytosine (5hmC) has emerged as a stable and functionally distinct epigenetic mark, separate from its initial identification as an intermediate in the process of active DNA demethylation. 5hmC is generated by the oxidation of 5-methylcytosine through the action of the ten-eleven translocation (TET) family of dioxygenase enzymes. These enzymes catalyze the stepwise conversion of methylated cytosines, which can lead to demethylation but also serve as stable modifications that influence gene regulation [43,44]. After fertilization, 5hmC is enriched in both paternal and maternal genomes, with preferential localization at enhancer regions, transcription factor binding sites, and genes essential for early development [45,46]. Recent single-cell analyses of human embryos revealed that 5hmC is deposited de novo on the maternal genome during oocyte maturation, persists through early embryogenesis, and contributes to lineage specification by localizing at key transcription factor binding sites such as OTX2 [45]. This emerging regulatory axis has been synthesized in recent comprehensive reviews emphasizing species-specific dynamics and diverse functional roles of 5hmC during germline development and early embryogenesis [47].

In the male germline, DNA methylation is erased in the primordial germ cells and re-established later in spermatogenesis, with methylation pattern establishment occurring prenatally and persisting through puberty [48,49]. The process results in the hypermethylation of most genomic areas except for certain developmental gene promoters and imprinted genes, which preserve the unique methylation patterns needed for proper embryonic development [50]. The correct regulation of such methylation patterns is crucial for sperm quality, fertilizing capacity, and embryonic development [48]. DNA methylation is distinct in the female germline. Genome-wide demethylation in primordial germ cells is followed by the establishment of female-specific patterns during oocyte growth and oocyte maturation [51]. In contrast to spermatogenesis, oocyte methylation is achieved postnatally during follicular development and is restricted to gene bodies rather than CpG islands [34]. These sex differences in methylation patterns reflect the male and female gametes’ distinct developmental trajectories and functions [52]. Olsen et al., (2021) reported that epigenetic regulation of granulosa cells leads to a reduced ovarian reserve, which may affect oocyte recruitment and growth [53]. Undergoing rapid active demethylation in fertilization and early embryo development, passively undergoing demethylation during subsequent cell divisions, paternal and maternal genomes combine to generate reprogramming, totipotency in early embryo formation, and lineage-specific differentiation [54,55,56]. Significantly, certain genomic areas, like imprinted genes, are exempted from this genome-wide reprogramming to maintain parent-of-origin-specific methylation signatures essential for normal development [57]. Interference in this process has been involved in several reproductive diseases and developmental abnormalities [58,59].

DNA methylation is essential for both the development and operation of male and female gametes by altering gene expression, genomic imprinting, and gametogenesis. In male germ cells, spermatogenesis depends on DNA methylation, which helps to properly silence transposable elements and create paternal imprinting marks required for embryonic development [60]. Irregularities in DNA methylation during spermatogenesis may lead to transgenerational epigenetic inheritance, infertility, or diminished sperm functionality [61]. DNA methylation facilitates X-chromosome inactivation, early embryonic development, and oocyte maturation in female reproductive cells [62]. Subpar oocyte quality, pregnancy loss, and developmental anomalies in progeny have been linked to alterations in oocyte DNA methylation patterns [63]. The preservation of fertility and embryonic viability over generations relies on the precise regulation of DNA methylation in reproductive cells. Dysregulations in DNA methylation during spermatogenesis may result in aberrant expression of target genes, potentially leading to infertility [64]. While most instances of idiopathic infertility may be attributed to underlying DNA methylation mechanisms, numerous epigenetic modifications leading to male reproductive failure remain unidentified [5]. Numerous genes exhibiting abnormal methylation have been linked to irregularities in semen parameters. Methylenetetrahydrofolate reductase (MTHFR) is one of the most extensively researched genes, serving as a crucial regulatory enzyme in folate metabolism, DNA synthesis, and methylation [65]. In both male and female gametes, the elimination and restoration of DNA methylation patterns are carefully controlled to guarantee appropriate epigenetic reprogramming, which is essential for early embryogenesis [66]. Infertility, miscarriage, and birth defects have all been linked to abnormal DNA methylation in reproductive cells, making reproductive health vital [6]. It is possible to learn about reproductive aging, fertility treatments, and possible epigenetic therapies by comprehending the mechanisms of DNA methylation in male and female germ cells [67].

## 4. Implications of Alteration in DNA Methylation

Alterations in DNA methylation disrupt gene expression and cellular function, resulting in various human diseases [68]. Specifically, abnormal gene expression has been linked to infertility in both males and females, as well as to reproductive potential and improper post-fertilization embryo development. Proper methylation of DNA is essential for the correct condensation of chromatin in the sperm head, which facilitates sperm maturation and enhances its capacity for fertilization and subsequent post-fertilization processes. Conversely, incomplete sperm chromatin condensation can lead to DNA damage, impairing the fertilization of egg cells and potentially reducing pregnancy rates [69]. In this context, some studies have examined the levels of gene and genome methylation in sperm DNA in relation to male reproductive dysfunctions [61,70]. Similarly, previous studies have elucidated that abnormal DNA methylation may significantly contribute to the development of conditions such as polycystic ovary syndrome (PCOS), endometriosis (EMS), and premature ovarian insufficiency, and that these defects in DNA methylation can lead to dysregulation of genes involved in immunity, and hormone synthesis [6,71,72]. Numerous abnormally methylated genes have been linked to altered sperm parameters, particularly oligozoospermia, suggesting a potential role of DNA methylation in male infertility [61]. Currently, well-established genetic causes of infertility in men with azoospermia and severe oligozoospermia include chromosomal anomalies, microdeletions in AZF regions, deletions on the long arm of the Y chromosome, and mutations in the CFTR gene [73]. Besides, various autosomal and sex-chromosomal genes are implicated in spermatogenic failure and male infertility [5]. While a significant proportion of male infertility remains idiopathic, the underlying molecular mechanisms are not yet fully elucidated. Furthermore, previous studies have reported abnormal DNA methylation in both imprinting (H19, IGF2, MEST, PEG3, LIT1, and SNRPN) and non-imprinting genes (MTHFR and DAZL) associated with various types of male infertility [31,74]. A study demonstrated that DMRs of imprinted genes, including SNRPN, MEG8, GNAS, and H19, revealed distinct methylation patterns in groups with abnormal semen [32], suggesting that alterations in DNA methylation within these genes are associated with abnormal semen parameters and compromised spermatogenesis.

### 4.1. Factors Responsible for Alteration in DNA Methylation

DNA methylation is a fundamental epigenetic modification that involves the addition of a methyl group to the fifth carbon atom of cytosine, primarily within CpG dinucleotides [75]. This dynamic process plays a critical role in regulating gene expression across the life course [75]. DNA methylation patterns are not static; they undergo profound reprogramming during gamete development and in early preimplantation embryos, and specific patterns are established and maintained throughout life [5]. While the genome provides the genetic blueprint, the epigenome acts as a reading rule, enabling precise gene expression regulation [75]. The epigenome can be altered by both internal factors, such as developmental processes and genetic background, and external factors, including environmental exposures, nutrition, stress, and lifestyle choices [76]. Understanding the interplay between genotype, age, health, nutrition, environment, and changes in the epigenotype is essential to identifying the unknown causes of infertility and other reproductive issues [76].

#### 4.1.1. Age and DNA Methylation

Advancing age profoundly influences the regulation of DNA methylation across both sexes—a process now widely recognized as epigenetic aging. This rapidly evolving field reveals that the accumulation of age-associated epigenetic modifications plays a pivotal role in shaping gene regulation, cellular function, and ultimately, organismal health [77,78,79,80]. In males, the aging process triggers extensive epigenetic reprogramming in sperm. DNA methylation patterns undergo significant modification, and complementary changes in histone marks and non-coding RNA profiles emerge with advancing age [81]. Although early investigations detected little evidence of global methylation shifts, more refined analyses have pinpointed discrete genomic regions exhibiting marked, age-dependent methylation changes [82,83]. Studies in fertile men consistently demonstrate that these epigenetic alterations occur at specific loci, reinforcing the notion that the aging process targets precise regions within the sperm epigenome [83]. Moreover, the development of sperm epigenetic clocks—predictive models based on DNA methylation signatures—further underscores the robust, non-random association between chronological age and targeted methylation changes [77,83,84]. The ramifications of these changes extend well beyond sperm quality. Age-induced epigenetic modifications in sperm have been directly linked to poorer reproductive outcomes. Anomalies such as deviations in the P1/P2 protamine ratio correlate with reduced IVF pregnancy rates and diminished fertilization capability [85], while concomitant chromatin remodeling underscores the extensive nature of these alterations [86]. More alarmingly, accumulating evidence connects advanced paternal age with the transmission of an altered methylome that may predispose offspring to neurodevelopmental disorders. Despite the near-complete demethylation of primordial germ cells during development, the paternal epigenetic signature endures in spermatozoa, suggesting that lifelong accrual of epigenetic changes carries transgenerational consequences [5,77,81,83,87].

In females, reproductive aging manifests through advanced maternal and post-ovulatory oocyte aging, severely compromising oocyte quality [78,80]. Investigations have documented age-related alterations in the DNA methylation landscape of oocytes. Given the critical role of DNA methylation in establishing genomic imprinting, a process essential for proper oocyte development and maturation, such epigenetic derangements have significant repercussions for fertilization efficiency and subsequent embryo development [78,80].

#### 4.1.2. Infection and DNA Methylation

**Figure 2:**
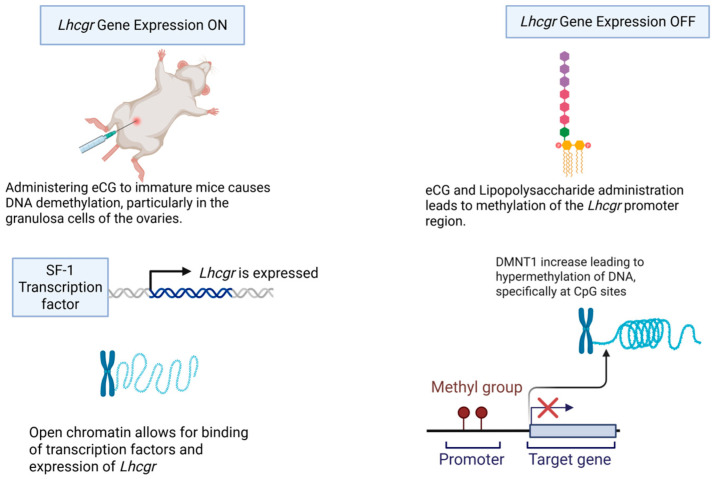
The role of infection in modulating DNA methylation.

Infections can impact DNA methylation in both males and females. In males, DNA methylation errors and changes in sperm miRNAs are associated with damage to sperm DNA integrity and spermatogenesis, which can be harmed by oxidative stress promoted by elevated inflammatory cytokines (such as IL-6 and IL-1β) released during infection [88]. Alterations in sperm DNA methylation patterns are linked to male infertility [89]. Perinatally acquired HIV has also been shown to alter DNA methylation in the peripheral blood of affected children, potentially leading to long-term health effects [90]. HIV infection can increase DNA methyltransferase activity in CD4+ T cells in vitro and is associated with altered DNA methylation in the host genome [91,92,93]. Notably, hypomethylation of two CpG sites in the promoter of NLRC5, a gene involved in immune response, is associated with HIV infection and negatively correlated with viral load [91]. In females, infections like Chlamydia trachomatis (CT) can induce epigenetic changes in genital tract epithelial cells that persist after the infection resolves [94,95,96]. This includes changes in DNA methylation, which can lead to a persistent epithelial-to-mesenchymal transition (EMT) phenotype [94,95,96]. CT infection has been linked to E-cadherin promoter methylation and the downregulation of E-cadherin expression [94], contributing to the EMT process that can result in fibrosis [94,95,96]. High-risk Human Papillomavirus (HPV) is also known to alter the host DNA methylome [93], and it is associated with cervical intraepithelial neoplasia and differential methylation of certain genes in women living with HIV [93]. Genital inflammation, often caused by STIs and bacterial vaginosis, is associated with an increased risk of HIV acquisition [88,97,98,99,100,101]. Furthermore, maternal infection and exposure to prenatal inflammatory signals can impact DNA methylation patterns in the offspring, as seen in studies on the hypothalamus of pigs, with potential long-term effects on processes like immune response and tissue development [90,102]. Bryan et al. (2021) investigated how chlamydial infection affects testicular cell lineages in mice, the infection led to substantial genomic fragmentation and altered gene expression in Leydig, Sertoli, and germ cells, interfering with key biological pathways, including interferon and germ-Sertoli cell signaling [103]. Moreover, the DNA in these cells and spermatozoa exhibited widespread hypomethylation. These genetic and epigenetic alterations are likely linked to subfertility in the infected mice and may contribute to birth defects in their offspring. Saki et al. showed that in spermatogenic cells exposed to *T*. *gondii*, a widespread protozoan parasite infecting warm-blooded mammals like mice and humans, there was a notable rise in the expression of DNMT1 and DNMT3A genes, which are crucial regulators of spermatogenesis [104]. Reproductive tract diseases can cause alterations in oocyte and endometrial methylation patterns in females. Baumann et al. (2015) show that endometriosis leads to significant epigenetic changes in the ovary, including altered expression of chromatin-remodeling enzymes like CARM1, PRMT2, and PRMT8 [105]. DNA hypermethylation of the PRMT8 promoter suggests that altered CpG methylation may repress gene expression, potentially affecting oocyte quality and contributing to infertility associated with endometriosis. Similarly, lipopolysaccharide-induced increase in the methylation rate of the *Lhcgr* promoter region in granulosa cells causes a decrease in the expression of *Lhcgr* and Cyp19a1, genes involved in ovulation and estrogen secretion [106]. Such infection-associated epigenetic alterations may lead to pelvic inflammatory diseases, infertility, habitual abortion, and adverse pregnancy outcomes reported in individuals with a history of reproductive tract infections [106,107] (Figure 2). Mechanisms by which infections induce these epigenetic changes are varied. Infections can alter DNA methylation directly or indirectly through inflammatory mediators [100]. Pathogens can influence host DNA methylation by inducing or repressing host DNA methylation enzymes (DNMTs and TETs) [100]. For example, HIV-1 infection can increase DNMT activity [100,108], while other bacteria can suppress DNMT activity or alter DNMT expression [100]. Some bacterial and parasitic pathogens may even express their own enzymes that function as methyltransferases, directly modifying the host epigenome [100,108,109]. The integration of foreign DNA, such as HIV-1 DNA, into the host genome can also lead to alterations in host methylation [91].

#### 4.1.3. Drugs and Steroids and DNA Methylation

Exposure to certain drugs and steroids can significantly alter DNA methylation patterns, impacting both male and female reproductive health and potentially influencing subsequent generations. Drugs such as anabolic androgenic steroids (AAS), recreational drugs, and some chemotherapy or immunosuppressive drugs have been associated with detrimental effects on the male reproductive system [110,111]. AAS abuse, for instance, induces testicular damage and interferes with testis development, function, and sperm characteristics, leading to decreased sperm count and motility [111,112,113,114]. The mechanism involves genetic and epigenetic factors, including changes in DNA methylation [111]. Studies indicate that 5α-dihydrotestosterone (a testosterone derivative) can increase DNA methylation in an animal model [111]. High-dose nandrolone exposure has also been linked to micronuclei formation, indicative of chromosomal damage, in testicle cells [111]. Epigenetic changes induced by certain lifestyle factors, such as cannabis use and exposure to endocrine-disrupting chemicals (EDCs), have been reported as potentially transferable to offspring, raising concerns about transgenerational effects [110]. Specific immunosuppressive drugs like sulfasalazine and cyclophosphamide have shown adverse effects on sperm quality, while methotrexate has a less clear impact [115]. Anabolic Androgenic Steroids (AAS), which are derivatives of testosterone, including nandrolone and boldenone, have been shown to affect reproductive tissues and induce epigenetic changes like DNA methylation alterations and micronuclei formation [111,116,117]. Testosterone supplements: Often associated with AAS abuse when used at supraphysiological doses [111,118,119]. The epigenetic impacts are similar to those described for AAS, affecting testicular structure and sperm parameters [111].

In females, exposure to hormonal contraceptives was determined to affect patterns of methylation of reproductive tissue. Koninckx et al. (2018) described that the extended duration of the usage of combined oral contraceptives resulted in effects on the methylation patterns within endometrial tissue, respectively affecting decidualization and implantation-related genes [120]. Similarly, Bunkar et al. (2016) and Doshi et al. (2011) proved that exposure of the oocytes during maturation and adult testis of rats by synthetic estrogens led to hypermethylation of the genes that were imprinted within mice oocytes and may have been able to influence embryonic growth and aberrant DNA methylation in testis [121,122]. Similarly, certain chemicals, particularly EDCs that mimic or interfere with hormones like estrogens, can induce epigenetic changes [110,123,124]. Studies involving estrogens and xenoestrogens, such as estriol (E3), diethylstilbestrol (DES), and bisphenol A (BPA), have demonstrated that exposure during fetal development can lead to altered gene expression and DNA methylation profiles in the adult female reproductive tract and brain [124,125,126,127]. For example, fetal E3 exposure altered global gene expression and methylation in the uterus and brain of offspring [124,125,126,127]. DES exposure in utero also resulted in permanent DNA methylation changes in the uterus by increasing DNA methyltransferases (DNMTs) level, enzymes crucial for maintaining methylation patterns [124,125,126,127]. These epigenetic modifications, programmed early in life, can result in long-term changes in gene expression and function [124,125,126,127].

Other chemical exposures with epigenetic impacts reported in previous studies include the fungicide propiconazole, which alters DNA methylation and DNMT expression in fish gonads and liver, leading to impaired reproduction and offspring development [123], and valproate, which induces replication-independent active DNA demethylation [128,129,130]. The processes through which drug-induced methylation modifications occur involve direct actions on DNMT function, changes in methyl donor pool, and oxidative stress-mediated DNA damage [131]. Such epigenetic changes can lead to reduced fertility and elevated developmental abnormality risk seen in the offspring of animals exposed to certain drugs and steroids [131,132].

#### 4.1.4. Impact of Stress and Lifestyle Factors

Psychological stress significantly impacts reproductive health, potentially through epigenetic modifications [133,134]. In men, stress activates the hypothalamic-pituitary-adrenal (HPA) axis, which can inhibit the hypothalamic-pituitary-gonadal (HPG) axis, reducing testosterone and suppressing spermatogenesis [133]. Perceived or occupational stress is linked to poorer semen quality, affecting sperm motility and morphology [133]. Paternal stress exposure can alter sperm DNA methylation, impacting stress-related genes and potentially affecting offspring stress response and behavior across generations in animal models [135,136]. Childhood abuse in men is also associated with changes in sperm DNA methylation [137]. For women, psychological stress and difficulty falling asleep are associated with an increased risk of infertility, potentially mediated by DNA methylation [71,138]. Maternal prenatal stress is linked to altered placental DNA methylation in stress-related genes (11-β-HSD2, NR3C1) and imprinted genes (e.g., IGF2, PEG3), affecting infant growth and birthweight [138].

Lifestyle factors, such as diet, obesity, smoking, alcohol intake, and lack of sleep, are recognized determinants of reproductive function and are linked to aberrant DNA methylation [133,139]. Diet significantly influences the control of DNA methylation in reproductive cells. Lambrot et al. (2013) discovered that folate deficiency in the father mouse caused the alteration of methylation patterning of sperm DNA, disrupting genes involved in the development, chronic disease, and metabolism in offspring [140]. Using sheep-model, maternal nutrition in early pregnancy, has been demonstrated to epigenetically modify offspring sperm DNA. Undernutrition altered DNA methylation at 240–244 sites, decreasing sperm motility and quality. Conversely, supplementation with folic acid provided a partial rescue, underlining the role of diet in transgenerational epigenetic inheritance [141]. Obesity has been a significant modulator of DNA methylation in reproductive cells. Donkin et al. (2016) reported that obese men possessed different sperm DNA methylation profiles than normal-weight men, affecting genes involved in controlling appetite and metabolism [142]. Similarly, Hou et al. (2016) demonstrated that obesity-induced alterations in oocyte DNA methylation affected genes crucial for embryonic development and placentation, reducing oocyte quality in obese mice [143]. Obesity in parents is associated with altered DNA methylation in genes related to metabolism and imprinting, potentially leading to metabolic disorders in offspring [139,144]. Cigarette smoking negatively affects sperm DNA methylation, increasing DNA damage and impacting semen parameters [145]. Smoking-induced methylation changes in sperm may contribute to increased health risks in offspring [145]. Poor sleep in men is linked to reduced testosterone and impaired sperm quality, while sleep alterations in women are associated with infertility, potentially mediated by DNA methylation [71]. Ecological toxicants, including endocrine-disrupting chemicals, heavy metals, and air pollutants, have been associated with aberrant methylation patterns in gametes. Iqbal et al. and Brieno-Enriquez et al. (2015) reported that endocrine-disruptor exposure caused hypomethylation of imprinted genes in sperm DNA, potentially affecting embryonic development and mature sperm [146,147]. Similarly, Manikkam et al. (2013) demonstrated that plasticizer exposure resulted in transgenerational alterations in sperm DNA methylation in rats, affecting genes involved in disease etiology [148]. The mechanisms underlying lifestyle-induced methylation include alterations in the availability of methyl donors, oxidative stress, endocrine disruption, and inflammation [149]. These epigenetic alterations may account for decreased fertility and increased risk of reproductive disease observed in individuals with unfavorable lifestyle factors [150]. Prenatal exposure to maternal cigarette smoking (PEMCS) has been noted to have dire consequences on fetal development because of critical DNA methylation changes that can be maintained into adulthood [151]. For instance, studies show that in the placenta, AluYb8 methylation level is higher in children exposed to cigarette smoke during the prenatal stage, causing changes in specific CpG methylation. Besides, methyl-specific ELISA studies have also shown global DNA hypomethylation in newborns from PEMCS and secondhand smoke exposure [152]. Nicotine exposure in the first trimester is associated with gene-specific DNA methylation changes in the lung and placenta [153]. These environment- and lifestyle-induced epigenetic alterations can impair gamete quality and have been associated with reduced success in assisted reproductive technologies (ART) [154]. Altered sperm DNA methylation is related to changes in semen parameters [155]. Furthermore, these epigenetic changes can be transmitted to offspring, increasing their predisposition to various health issues [135]. Modifying lifestyle may help alleviate any adverse effects on reproductive potential [156] (Table 1).

## 5. Conclusions and Future Perspectives

DNA methylation is the most extensively studied epigenetic regulation and has been linked to reduced reproductive potential in males and females and abnormal post-fertilization embryo development. Notably, aberrant DNA methylation frequently influences reproductive outcomes. Therefore, discussing specific genes affected by abnormal DNA methylation and factors such as steroids, drugs, age, infection, and lifestyle influencing DNA methylation is essential for understanding infertility issues and elucidating the precise regulators of gametogenesis and embryogenesis. This understanding will enhance clinical assessment of couples facing infertility challenges and uncover novel therapeutic targets for treatment.

In further context related to reproductive outcomes, numerous studies have contributed substantial evidence to the hypothesis that sperm methylation is associated with sperm alterations and infertility. Specifically, studies have examined the relationships between abnormal DNA methylation in certain genes with irregular sperm parameters, such as count, concentration, morphology, and/or motility. In spite of these efforts, the critical role of improper DNA methylation marks in inducing male infertility remains largely underexplored, particularly due to the limited studies on the mechanisms of DNA methylation in sperm cells. It is plausible that aberrations in methylation at specific target genes may indicate broader methylome defects resulting from altered DNMT activity during sperm cell development and spermatogenesis. Therefore, there is a pressing need for new functional studies to clarify the mechanisms that influence methylation in sperm. Furthermore, a comprehensive understanding of sperm DNA methylation status in relation to diminished reproductive capability is valuable for the development of innovative diagnostic tools for infertility.

## Figures and Tables

**Table 1:** Various endogenous and environmental factors that influence DNA methylation.

Factor	Genes/Enzymes/Pathways Affected	References
Age	*DENND1A*, *TCF20*, *HOXD8*	[78,80]
Infection	NLRC5, E-cadherin, *Lhcgr*, Cyp19a1, IL-6 and IL-1β, like CARM1, PRMT2, and PRMT8	[88,91,94,105,106]
Drugs/Steroids	FSH and LH	[114]
Stress	11-β-HSD2, NR3C1IGF2, PEG3	[138]
Obesity	BDNF, FTO, SH2B1, CHST8	[142]
Alcohol intake and Smoking	lipid peroxidation (MDA) and EAO which included catalase (CAT), superoxide dismutase (SOD) and glutathione reductase (GR)	[110]
Chemical Exposure/Toxicants	HOXA10, MTHFR, imprinted genes (IGF2, PEG3)	[146,147]

## Data Availability

Not applicable.
