# Correction: Adetunji et al. DNA Methylation: A Key Regulator in Male and Female Reproductive Outcomes. *Life* 2025, *15*, 1109

**DOI:** 10.3390/life15121898

**Published:** 2025-12-12

**Authors:** Adedeji O. Adetunji, Henrietta Owusu, Esiosa F. Adewale, Precious Adedayo Adesina, Christian Xedzro, Tolulope Peter Saliu, Shahidul Islam, Zhendong Zhu, Olanrewaju B. Morenikeji

**Affiliations:** 1 Department of Agriculture, University of Arkansas at Pine Bluff, Pine Bluff, AR 71601, USA; owusuh8210@uapb.edu (H.O.); islams@uapb.edu (S.I.); 2 Department of Biology, University of Louisville, Louisville, KY 40292, USA; esiosa.adewale@louisville.edu; 3 Graduate School of Integrated Sciences for Life, Hiroshima University, 1-4-4 Kagamiyama, Higashihiroshima 739-8528, Japan; precious.adesina@nih.gov; 4 Laboratory of Food Microbiology and Hygiene, Hiroshima University, Higashihiroshima 739-8528, Japan; xedzro@hiroshima-u.ac.jp; 5 Department of Physiology, College of Medicine, University of Kentucky, Lexington, KY 40536, USA; tpsa222@uky.edu; 6 College of Animal Science and Technology, Qingdao Agricultural University, Qingdao 266109, China; zzd2020@qau.edu.cn; 7 Department of Biological and Health Sciences, University of Pittsburgh, Bradford, PA 16701, USA; obm3@pitt.edu

In the published publication [1], there was an error regarding the affiliation for Precious Adedayo Adesina. The updated affiliation should be as follows: Graduate School of Integrated Sciences for Life, Hiroshima University, 1-4-4 Kagamiyama, Higashihiroshima 739-8528, Japan. The authors state that the scientific conclusions are unaffected. This correction was approved by the Academic Editor. The original publication has also been updated.
